# Presbyopia of Civilization

**Published:** 1902-02

**Authors:** 


### Presbyopia of Civilization.

In discussing the presbyopia of civilization, *New York Medical Record,* Dr. Norburne B. Jenkins, of Chicago, says: "In some parts of civilization the habits and customs of life weaken, injure and prematurely age the eyes. After correcting with distant-vision tests the total imperfections of the size and shape of the eyeballs, in Boston most people about forty years of age need magnifying glasses for reading; in New York, most after forty-two need such, and in Chicago at about forty-five, while in the mountains, plains and farming country of the South and Middle West such help is not needed until about the fiftieth year. Indoor life, air starvation, insufficient fresh meat, long hours in close schools, lack of play, vegetarianism, unsanitary houses and clothes, non-use of the eyes for long distances, lack of green fields and forests, short, dangerous distances of the school room, powerful artificial lights, unnatural surroundings of overprotected and finicky city life—all tend to ruin the eyes." R.

